# Estimating the effects of reopening of schools on the course of the epidemic of COVID-19

**DOI:** 10.1017/S0950268821000686

**Published:** 2021-04-05

**Authors:** Eduardo Massad, Marcos Amaku, Dimas Tadeu Covas, Luis Fernandes Lopez, Francisco Antonio Bezerra Coutinho

**Affiliations:** 1 School of Medicine, University of Sao Paulo and LIM01-HCFMUSP, Sao Paulo, Brazil; 2 School of Applied Mathematics, Fundacao Getulio Vargas, Rio de Janeiro, Brazil; 3 School of Veterinary Medicine, University of Sao Paulo, Sao Paulo, Brazil; 4 Instituto Butantan, Sao Paulo, Brazil

**Keywords:** COVID-19, mathematical modelling, school children

## Abstract

In this paper, we present a method to estimate the risk of reopening of schools illustrated with the case of the State of São Paulo, Brazil. The model showed that, although no death of children would result from the reopening of the schools in the three cities analysed, the risk of asymptomatic and symptomatic cases and secondary cases among teachers, school staff and relatives of the children is not negligible. Although the epidemic hit different regions with different intensities, our model shows that, for regions where the incidence profile is similar to the cities analysed, the risk of reopening of schools is still too high. This in spite of the fact that incidences in these cities were declining in the period of the time considered. Therefore, although we cannot extend the result to the entire country, the overall conclusion is valid for regions with a declining incidence and it is even more valid for regions where incidence is increasing. We assumed a very conservative level of infection transmissibility of children of just 10% as that of adults. In spite of the very low level of transmissibility is assumed, the number of secondary cases caused by infected children among teachers, school staff and relatives varied from 2 to 85. It is, therefore, too soon to have any degree of confidence that reopening of schools before the advent of a vaccine is the right decision to take. The purpose of our model and simulations is to provide a method to estimate the risk of school reopening, although we are sure it could be applied as a guide to public health strategies.

## Introduction

It is estimated that since the beginning of the COVID-19 pandemic early in 2020, 90% of students globally have had their education severely disrupted by the mitigation procedures [1]. This means that approximately 1.6 billion students have been out of school worldwide as a result of the global lockdown [2]. Currently, although some places refrained from closing schools, the majority of countries are battling over whether and how to reopen schools.

There is now emerging evidence about the consequences of school closures on children education, social development relationships, mental and physical wellbeing and exposure to violence, in addition to decreasing access to support services including meals [3]. In addition, it is now acknowledged that schoolchildren play a relatively small role in the transmission of the coronavirus [4], and are less likely to be symptomatic [5]. Moreover, it has been estimated that only around 2–4% of COVID-19 deaths were prevented as a result of school closures [6]. Notwithstanding these facts, studies suggest that reopening of schools could lead to second waves of infection [7] and that the impact of reopening of schools is expected to vary across a range of European countries [8].

Therefore, the challenges that government face on deciding whether and how to safely reopen schools are determined by a balance between the potential benefits of returning to schools and the risks of infection, to children, teachers and school staff and to societies at large [4].

Brazil is among the 191 countries that decided to close schools to mitigate the impact of the coronavirus in March 2020 and the decision to reopening of schools is now being discussed around the country by local State and Municipal authorities.

The State of São Paulo, the most populous and most affected by the COVID-19 outbreak is planning a gradual reopening of schools. The reopening of schools in the spring of 2020 in the state of Sao Paulo seems to be both inevitable and perhaps desirable. Although there is some evidence that the effect may not be so great as feared before, some effect is expected. It is, therefore, useful and instructive to use mathematical models to estimate this effect.

To this end, we choose three cities in the State of Sao Paulo, small enough for the model to work well and to illustrate the estimation of the impact of reopening of schools on the epidemic course.

In the next section, we describe the model used and its limitations. Then, in the section ‘Calculating the incidence of the infection’, we show how to take advantage of present reported incidence to estimate the parameters of the model. In the section ‘The cities analysed’, we describe three cities chosen and the data collected from them. In section ‘Results’, we describe our results, and in the section ‘Discussion’, we discuss our findings. In the sensitivity section we describe further details of the results of a sensitivity analysis of the relative transmissibility of the virus in children as related to adults.

## The model

The model is a modified version of the classical SEIR type of models [9, 10] and considers that the total population involved is divided into:

1. Susceptible individuals, denoted *S*(*t*), die by natural causes with rate *μ*, or acquire the virus with a contact rate *β*.
2. Once infected, the susceptible moves to the state of exposed, denoted *E*(*t*). These individuals either die by natural causes with the same rate *μ*, or evolve to the infectious individuals, denoted *I*(*t*), with rate a *δ*_I_, or evolve to asymptomatic/oligosymptomatic individuals, denoted *A*(*t*), with rate *δ*_A_.
3. Infectious individuals, *I*(*t*), either die by natural causes with a rate *μ*, or by the disease, with rate *α*_I_, or recover from the infection to a new state, denoted *R*(*t*), with rate *γ*_I_.
4. Individuals in the state*A*(*t*) can die by natural causes with rate *μ*, or recover with a rate *γ*_A_.

We assume that the population birth rate Λ(*t*) is equal to the natural mortality of the population, not taking into account the disease-induced mortality.

**Fig. 1.**
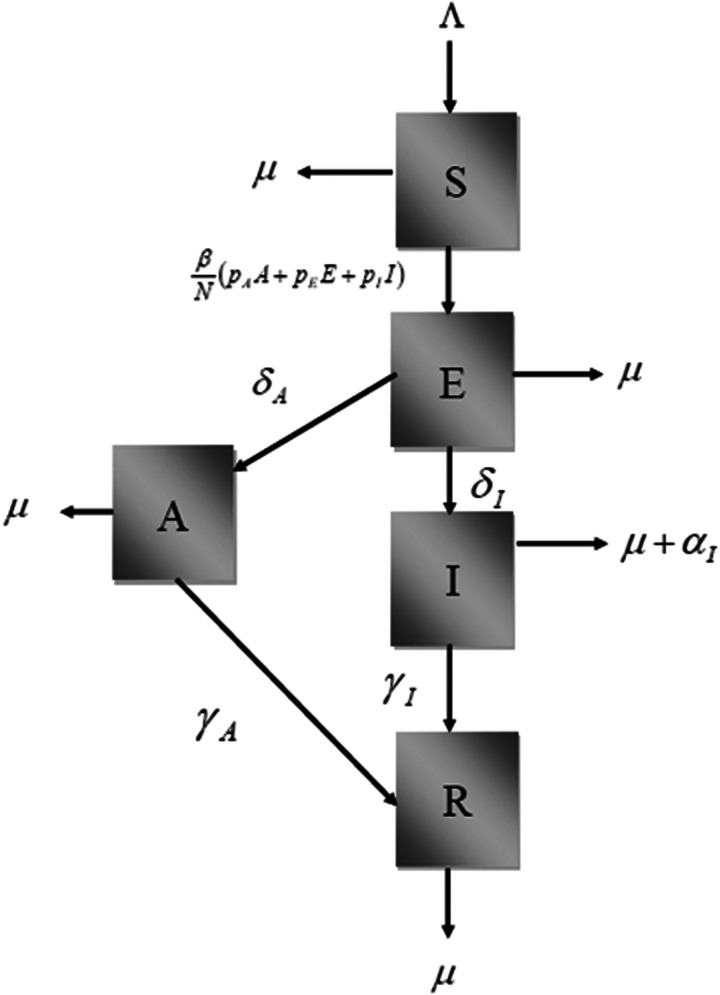
Diagram showing the model's compartments and transitions.

The states of the model are shown in Figure 1.

The dynamics of the model is described by the following set of differential equations:1
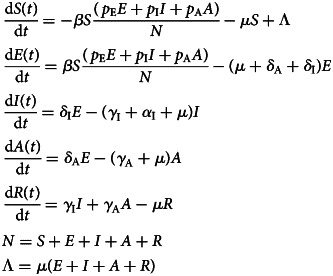


The basic reproduction number of system (1) is given by:2
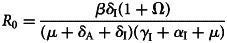
where3
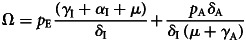


The incidence of infection is given by:4
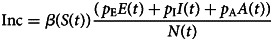


The total number of infections is obtained by:5
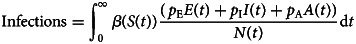


The total number of clinical cases is obtained by:6
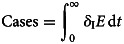


The total number of exposed individuals is given by:7
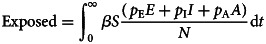


Finally, the total number of COVID-19-related deaths is given by:8
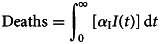


## Calculating the incidence of the infection

The daily incidence of each city, as reported by the health authorities, was fitted to a continuous function with the form:9
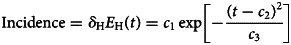
where *c*_*i*_  (*i* = 1, …, 3) are the fitting parameters and *t* is the time.

From the empirical data on the daily incidence, it is possible, from equation (4) to estimate the potentially infective contact rate *β*(*t*), such that:10
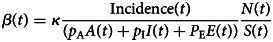


We considered that children are 90% less infective than adults (15) and therefore, equation (10) was divided by 10 (that is, *κ* = 0.1). In the ‘Results’ section, we show a sensitivity analysis of the parameter *β*(*t*) to the children transmissibility parameter *κ*.

Finally, the model assumes similar virulence and transmissibility for the three cities analysed, which, in the absence of empirical evidence to the contrary, seems to be a reasonable assumption.

## The cities analysed

**Fig. 2.**
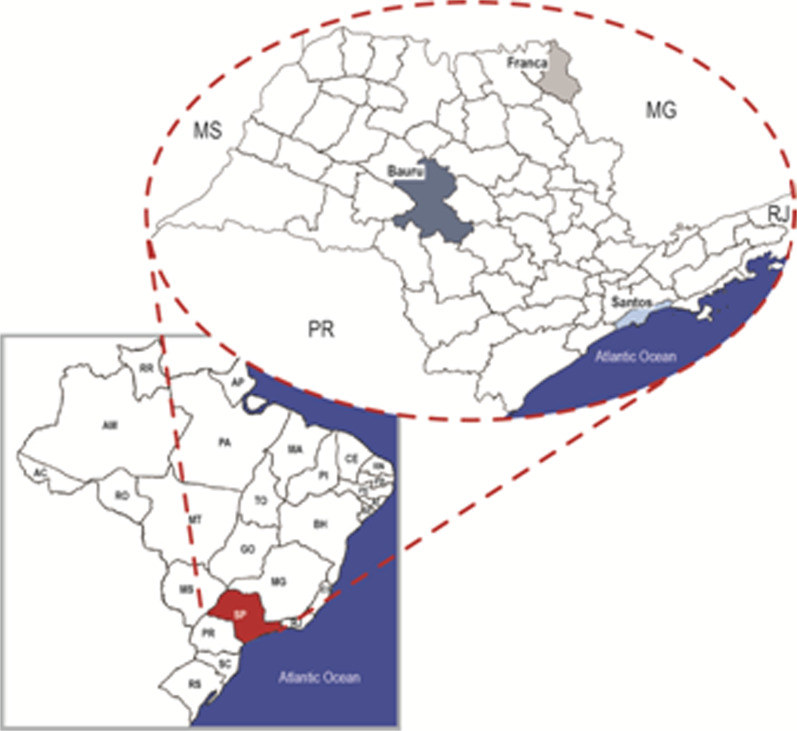
Map showing the localisation of the three cities analysed.

To estimate the impact of school reopening at the current stage of the COVID-19 outbreak in Brazil, we chosen (choose?) three cities of the State of São Paulo, which are approximately of the same size and from which detailed information of the daily incidence of the infection is available. They are the city of Santos (433 656 inhabitants), Bauru (379 297 inhabitants) and Franca (355 901 inhabitants). Figure 2 shows the localisation of the three cities.

Table 1 shows the characteristics of the cities related to the current study.

**Table 1.** Demographic and epidemiological characteristics of the cities analysed

City	Pop. 0–9 years of age	Reported cases of infection	Reported deaths	Estimated number of infected children (see text)
Santos	43 809	22 767	639	400
Bauru	43 225	13 379	240	258
Franca	44 064	6152	183	129

Note that the population aged from 0 to 9 years, the age interval of interest for this study, is remarkably similar in the three cities.

The estimated number of actively infective children was calculated as follows. First, we assumed that children from 0 to 9 years old represent 2.5% of the reported cases [11] to obtain the number of reported cases in this age strata. Then, we multiplied the officially reported number of cases by 7 (estimated by seroprevalence result in [12]) to obtain the total number of infectious individuals. This total was multiplied by 0.12 to account for the fact that 88% of the cases have recovered [13]. This gives the total number of currently active infections. The latter was then multiplied by 0.025, the observed average prevalence in children observed in the State of São Paulo, to obtain the estimated number of actively infectious children in each city. We also assumed that disease-induced mortality of children was equal to the average 0.1% of cases as observed in the State of São Paulo.

## Results

**Fig. 3.**
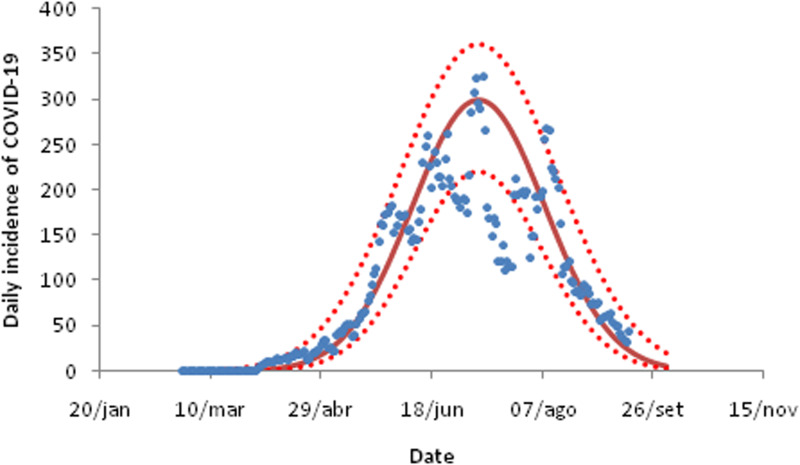
Fitting the daily incidence of COVID-19 to the data from the city of Santos, São Paulo to equation (8). Dots are actual data, continuous line the average curve and dotted curves the 95% CI.

**Fig. 4.**
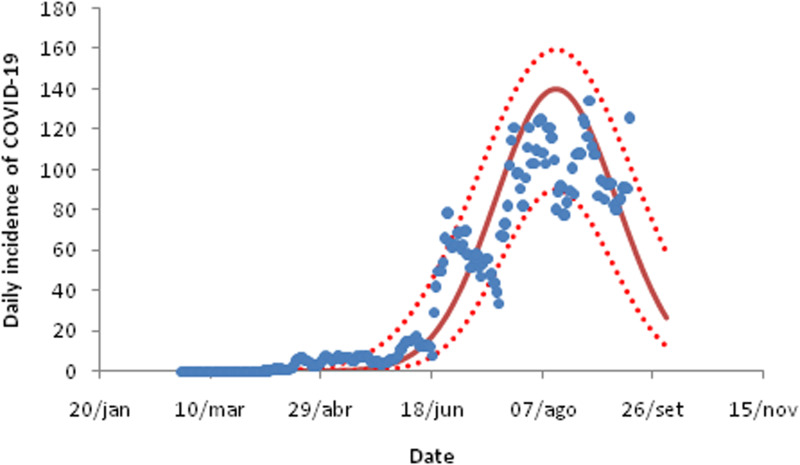
Fitting the daily incidence of COVID-19 to the data from the city of Bauru, São Paulo to equation (8). Dots are actual data, continuous line the average curve and dotted curves the 95% CI.

**Fig. 5.**
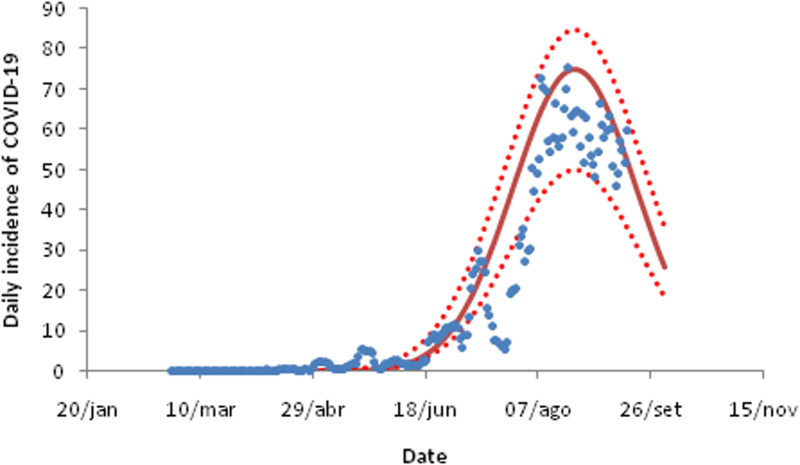
Fitting the daily incidence of COVID-19 to the data from the city of Franca, São Paulo to equation (8). Dots are actual data, continuous line the average curve and dotted curves the 95% CI.

For the three cities in the state of São Paulo analysed in this study, the daily incidence of COVID-19 fitted to equation (9) are shown in Figures 3–5. The fitting parameters are shown in Table 2 (the numbers in brackets represent the 95% confidence intervals (CIs)).

**Table 2.** Fitting parameters to the incidence of infection in each city (95% CI)

City	*C*_1_	*C*_2_	*C*_3_
Santos	302 (221–363)	135 (128–141)	1805 (1655–2507)
Bauru	144 (93–162)	173 (168–173)	1507 (1251–2508)
Franca	75 (51–85)	182 (177–183)	1610 (1509–1848)

Model (1) was numerically simulated with parameters as shown in Table 3.

**Table 3.** Parameters used in the simulations of model (1)

Parameters	Biological meaning	Value^a^
*β*(*t*)	Potentially infective contact rate	From equation (10)
*p*_E_	Infectivity of exposed	0.4
*p*_I_	Infectivity of infected (cases)	1.0
*p*_A_	Infectivity of asymptomatic	0.07
*μ*	Natural mortality rate	3.91 × 10^−5^ per day
*δ*_I_	Rate of evolution from exposed to infected	8.0 × 10^−2^ per day
*δ*_A_	Rate of evolution from exposed to asymptomatic	5.0 × 10^−2^ per day
*γ*_I_	Rate of recovery from infected	3.3 × 10^−1^ per day
*γ*_A_	Rate of recovery from asymptomatic	7.0 × 10^−2^ per day
*α*_I_	Disease-induced mortality rate^b^	2.0 × 10^−4^ per day


a Parameter values are from [10].

b We assumed a case fatality rate that corresponds to 0.1% of the total deaths in São Paulo (SEADE, 2020).

The results of the numerical simulations of model (1) for each of the cities studied are shown in Tables 4–6 where numbers refer to cases attained after 10, 20 and 30 days.

**Table 4.** Results of the simulations for the city of Santos (95% CI)

Day	Asymptomatics	Symptomatics	Deaths	*R*_eff_(*t*)	Secondary cases
0	0	0	0	0.06 (0.02–0.17)	0
10	107 (101–126)	17 (16–20)	0	0.20 (0.061–0.62)	6 (2–27)
20	114 (104–153)	18 (17–24)	0	0.23 (0.066–0.71)	8 (2–44)
30	117 (104–168)	19 (17–27)	0	0.17 (0.04–0.64)	6 (1–46)

**Table 5.** Results of the simulations for the city of Bauru (95% CI)

Day	Asymptomatics	Symptomatics	Deaths	*R*_eff_(*t*)	Secondary cases
0	0	0	0	0.24 (0.11–0.39)	0
10	28 (9–63)	14 (12–17)	0	0.81 (0.39–1.25)	28 (9–63)
20	28 (12–114)	18 (17–26)	0	0.86 (0.43–1.32)	28 (12–4)
30	43 (10–139)	20 (14–32)	0	0.73 (0.33–1.25)	43 (10–39)

**Table 6.** Results of the simulations for the city of Franca (95% CI)

Day	Asymptomatics	Symptomatics	Deaths	*R*_eff_(*t*)	Secondary cases
0	0	0	0	0.37 (0.21–0.49)	0
10	52 (43–60)	8 (7–10)	0	1.14 (0.77–1.39)	27 (12–42)
20	74 (54–95)	12 (9–15)	0	1.15 (0.85–1.40)	43 (20–71)
30	87 (34–115)	14 (10–18)	0	1.00 (0.74–0.27)	47 (21–85)

The tables show the number of asymptomatic and symptomatic children, the number of deaths by COVID-19, the effective reproduction number and the secondary cases of infection caused by infected children. The effective reproduction number, *R*_eff_(*t*), was calculated according to the equation:11
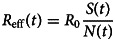


The estimated number of secondary cases among teachers, school staff and children's cohabitants caused by infected children was calculated by the equation:12
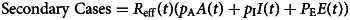


We simulated the school reopening on 16th September for the period of 30 days. As shown in Tables 4–6, day 0 is the date of the beginning of the simulations. We show the results attained at days 10, 20 and 30.

It can be noted that, in spite of the fact that no deaths would result from the reopening of the schools, the number of asymptomatic cases varied from the minimum of 9 (inferior confidence interval at day 10 in Bauru) to the maximum of 168 (superior confidence interval at day 30 in Santos); the number of symptomatics varied from the minimum of 7 (inferior confidence interval at day 10 in Franca) to the maximum of 32 (superior confidence interval at day 30 in Bauru); but more concerning is the number of secondary cases caused by infected children among their teachers, school staff and cohabitants which varied from the minimum of 2 (inferior confidence interval at day 10 in Santos) to the maximum of 85 (superior confidence interval at day 30 in Franca).

## Sensitivity analysis

**Fig. 6.**
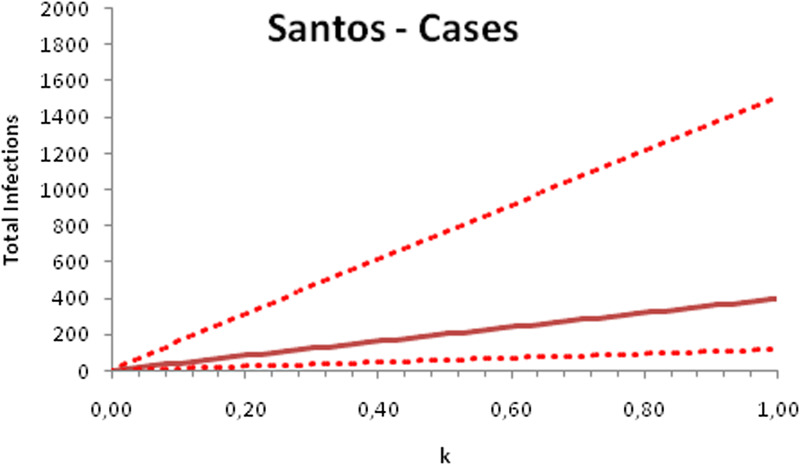
Total number of infections after 30 days of school reopening as a function of the transmissibility parameter *κ*. Continuous line represents the average curve and dotted lines the 95% CI.

**Fig. 7.**
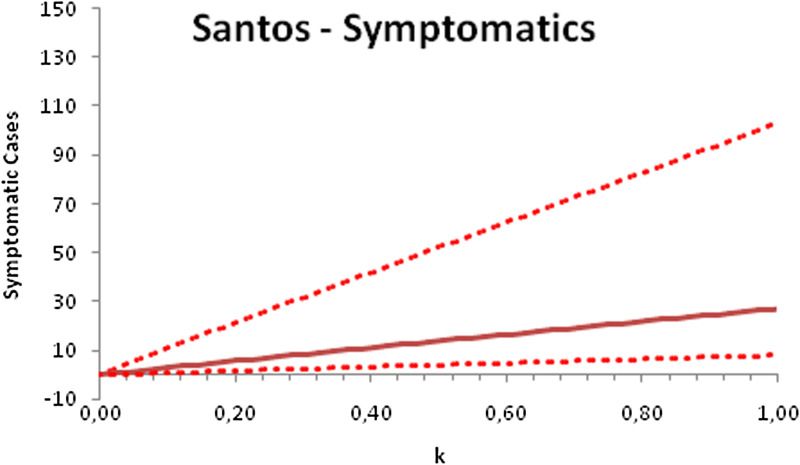
Total number of symptomatic children after 30 days of school reopening as a function of the transmissibility parameter *κ*. Continuous line represents the average curve and dotted lines the 95% CI.

**Fig. 8.**
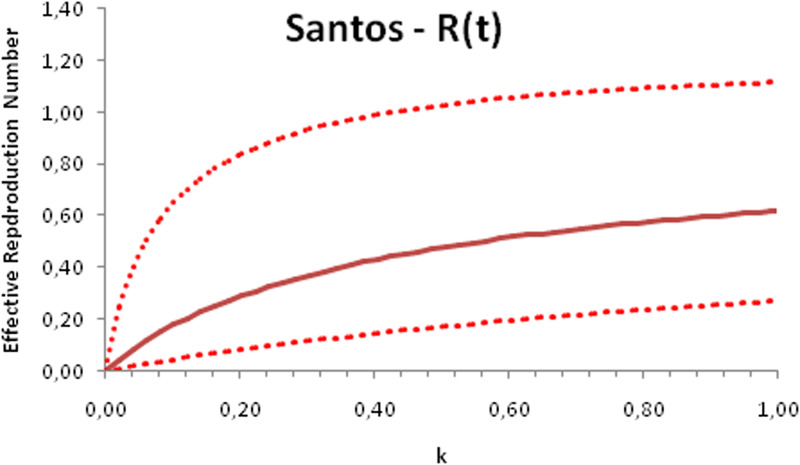
Effective reproduction number after 30 days of school reopening as a function of the transmissibility parameter *κ*. Continuous line represents the average curve and dotted lines the 95% CI.

**Fig. 9.**
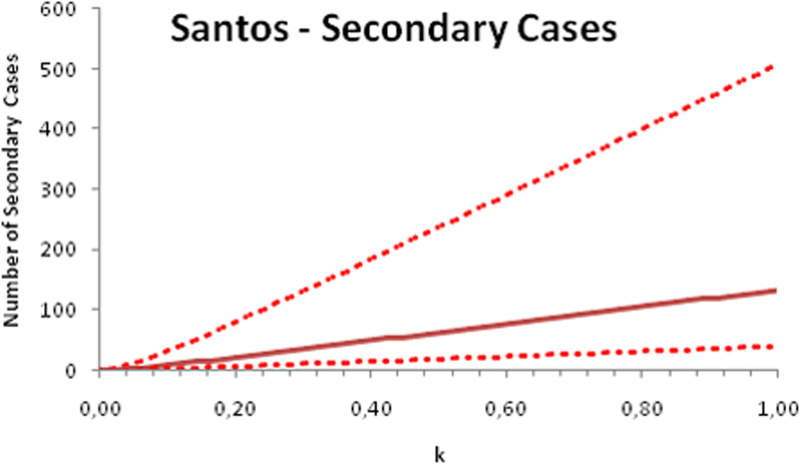
Total number of secondary infections after 30 days of school reopening as a function of the transmissibility parameter *κ*. Continuous line represents the average curve and dotted lines the 95% CI.

**Fig. 10.**
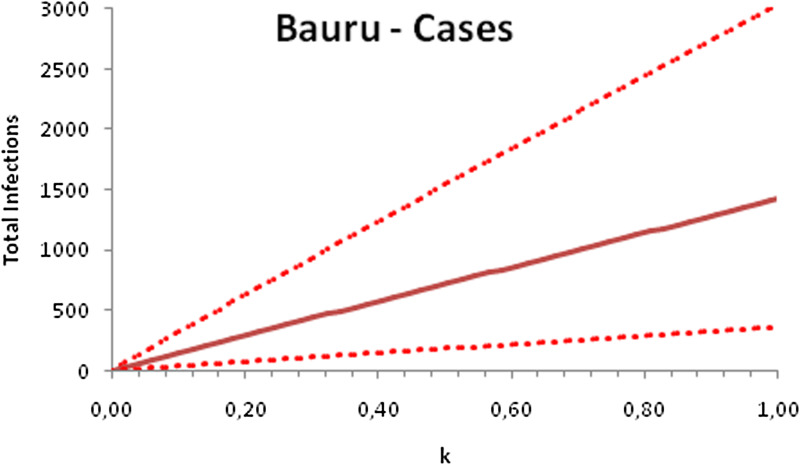
Total number of infections after 30 days of school reopening as a function of the transmissibility parameter *κ*. Continuous line represents the average curve and dotted lines the 95% CI.

**Fig. 11.**
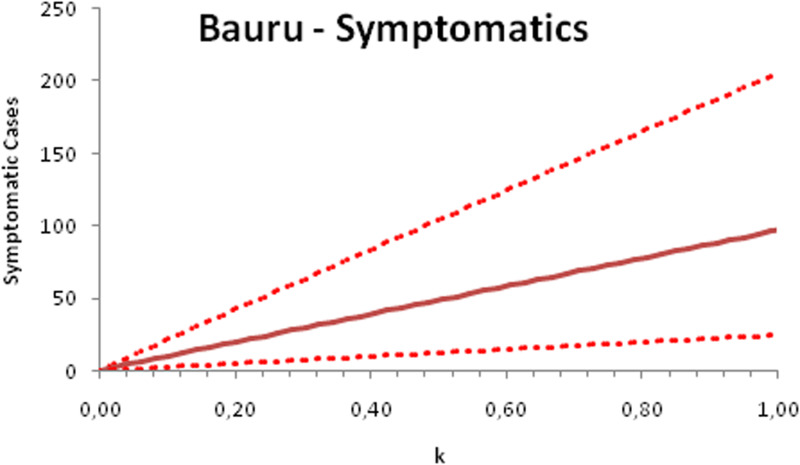
Total number of symptomatic infections after 30 days of school reopening as a function of the transmissibility parameter *κ*. Continuous line represents the average curve and dotted lines the 95% CI.

**Fig. 12.**
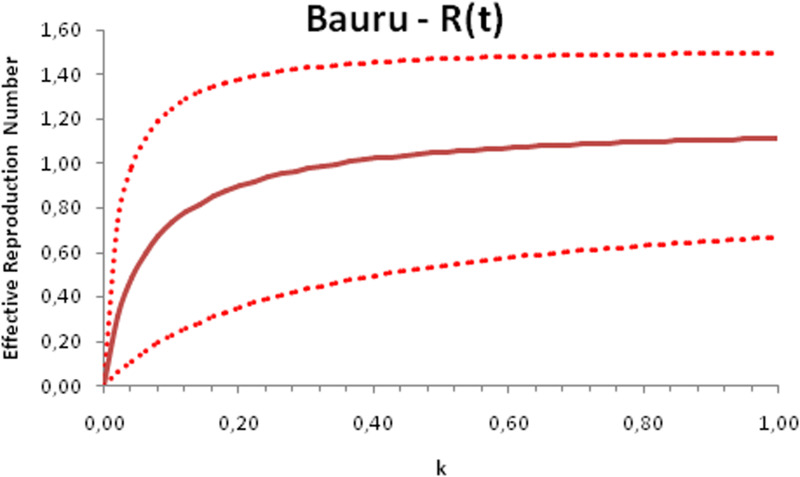
Effective reproduction number after 30 days of school reopening as a function of the transmissibility parameter *κ*. Continuous line represents the average curve and dotted lines the 95% CI.

**Fig. 13.**
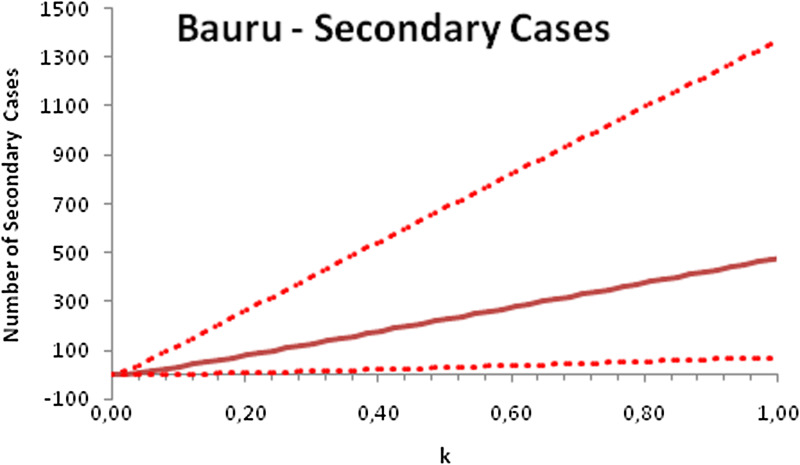
Total number of secondary infections after 30 days of school reopening as a function of the transmissibility parameter *κ*. Continuous line represents the average curve and dotted lines the 95% CI.

**Fig. 14.**
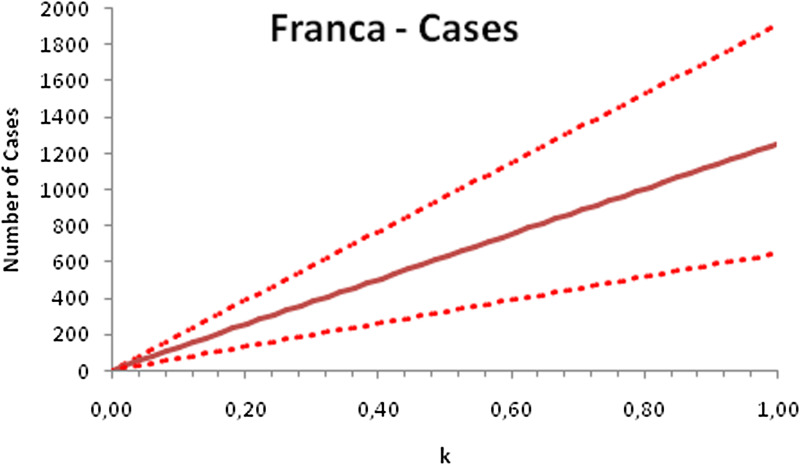
Total number of infections after 30 days of school reopening as a function of the transmissibility parameter *κ*. Continuous line represents the average curve and dotted lines the 95% CI.

**Fig. 15.**
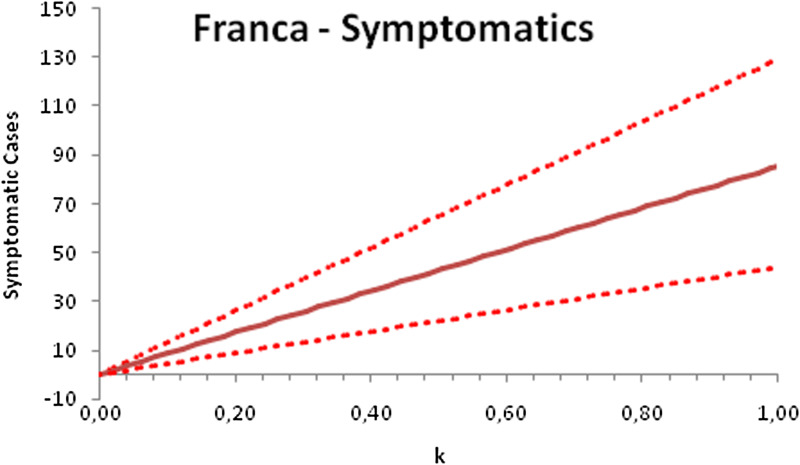
Total number of symptomatic infections after 30 days of school reopening as a function of the transmissibility parameter *κ*. Continuous line represents the average curve and dotted lines the 95% CI.

**Fig. 16.**
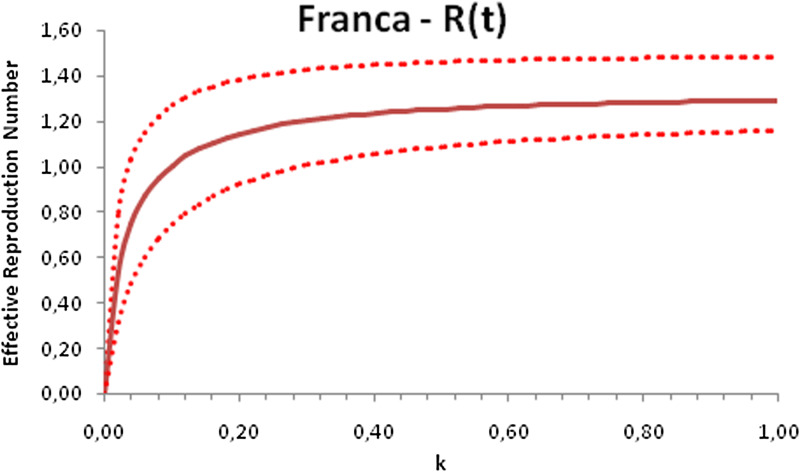
Effective reproduction number after 30 days of school reopening as a function of the transmissibility parameter *κ*. Continuous line represents the average curve and dotted lines the 95% CI.

**Fig. 17.**
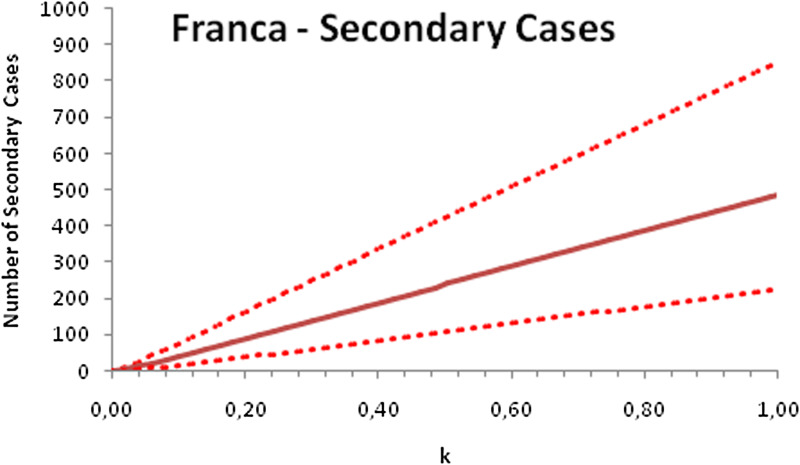
Total number of secondary infections after 30 days of school reopening as a function of the transmissibility parameter *κ*. Continuous line represents the average curve and dotted lines the 95% CI.

In this section, we show a sensitivity analysis of the parameter *β*(*t*) to the children transmissibility parameter *κ*, the relative transmissibility of children with respect to adults. We varied the parameter *κ* from 0 (no transmissibility) to 2 (double the transmissibility of adults) and numerically calculated the total number of infections (cases), number of symptomatics, the effective reproduction number and the number of secondary infections of school staff and cohabitants caused by children after 30 days of the reopening of schools. We do not show the mortality results because the model show no deaths in none of the cities among children, even when the assumed transmissibility was equal to 2, that is, the case when children were considered twice as much transmissible as adults. The results are shown in Figures 6–17.

With the exception of the effective reproduction number, it can be noted that the analysis of sensitivity for the three cities shows that all the other variables vary linearly with the transmissibility parameter *κ*.

## Discussion

In this paper, we present a method do estimate the risk of reopening of schools in the middle of an outbreak of COVID-19, illustrated by the case of the State of São Paulo, Brazil.

For the three cities analysed, the model show that although no death of children would result from the reopening of the schools, the risk of symptomatic cases and secondary cases among teacher, school staff and relatives of the children is not negligible.

We assumed a very conservative level of infection transmissibility of children of just equal to 10% of adults. This is consistent with the well-established fact that children are less likely to develop severe disease from COVID-19 compared to adults [14–16]. Even with this assumed very low level of transmissibility, the number of secondary cases caused by infected children among teachers, school staff and relatives varied from 2 to 85.

As the actual transmissibility of SARS-CoV-2 of children as compared to adults is not known with any level of certainty, we decided to carry out a sensitivity analysis. We observed a linear relationship between the parameter *κ*, expressing the relative transmissibility and the number of total, symptomatic and secondary infections. The effective reproduction number, however, showed a highly non-linear relationship with parameter *κ*. Another important finding is that no deaths in the first 30 days of school reopening resulted from the simulations of the model. Moreover, the sensitivity analyses showed that if children's transmissibility was equal to the adults, an upper limit for the city of Bauru could reach 1500 secondary cases caused by infected children among their teachers, school staff and cohabitants.

It is noteworthy that, although the epidemic hit different regions with different intensities, our model shows that, for regions where the incidence profile is similar to the cities analysed, the risk of reopening of schools is still too high. This in spite of the fact that incidences in these cities were declining in the period of the time considered. Therefore, although we cannot extend the result for the entire country, the overall conclusion is valid for regions with a declining incidence and it is even more valid for regions where incidence is increasing.

Also important is the fact that the model does not take into account the transmission between teachers and school staff. We are interested in the transmission between children and how infected children would infect teachers, school staff and children's relatives. Transmission among teachers and school staff would be a posterior phenomenon and would be a natural consequence of the transmission chain triggered by infected children. Of course, an infected teacher could also trigger a transmission sequence to children, but this has not been considered in the present model.

The Government of the State of São Paulo has authorised the partial opening of schools since September 2020 and, so far, only 16 cases of COVID-19 have been reported [17]. However, it should be pointed that the schools in the State of Sao Paulo were only very partially reopened, with approximately 25% of children attending classes face-to-face. It should be, therefore, expected that the number of cases would be lower than the ones reported in this paper. In our simulations, the schools were assumed fully opened, with 100% of attendance.

It should be stressed that the purpose of our model and simulations is to provide a method to estimate the risk of school reopening, although we are sure it could be applied as a guide to public health strategies. Notwithstanding, by assuming an extremely low level of transmissibility of just 10% of that of adults, our results give some support to the conclusions reached by Sheik *et al*. [1] that schools should remain closed until a vaccine can be administered at sufficient levels to achieve herd immunity or a treatment is found. In addition, the effective reproduction number should be well below unity. A viable alternative is to partially reopen schools, such that there are fewer students at school simultaneously to enable proper social distance. This is the strategy adopted so far by the State of Sao Paulo. In any case, surveillance based on testing and isolating positive cases is crucial to avoid the spread of the virus to other class mates, teachers, school staff and relatives of the children.

An important limitation of our model is that it is not individual-based model and therefore, there is no contact matrix. However, as we assumed the incidence function as fitted to actual data as the main input of the model, the transmission is implicit in that function.

Finally, the State of São Paulo reported a significant uptick in the daily incidence of COVID-19 in the last week (from 9 to 16 November 2020). If this is a signal of a second wave of the pandemic, this means an additional reason to postpone the decision of reopening of the schools until a safe and effective vaccine is available, or natural herd immunity is achieved. It is, therefore, too soon to have any degree of confidence that reopening of schools before the advent of an effective vaccine is the right decision to take, notwithstanding the long-term psychological, social and economic consequences of schools’ closure. Of course, there are safe alternatives to full closure of schools, such as restricting the number of students, guarantee of proper ventilation of classes, staging different opening and closing times, including break periods during the days and other mitigation measures. However, in situations where the incidence of new cases in the community is greater than 5% per day, opening school should be considered with great care.

## Data Availability

All published findings, such as data, code and other materials, are available to readers without undue barriers to access.
